# An Electrochemical NO_2_ Sensor Based on Ionic Liquid: Influence of the Morphology of the Polymer Electrolyte on Sensor Sensitivity

**DOI:** 10.3390/s151128421

**Published:** 2015-11-11

**Authors:** Petr Kuberský, Jakub Altšmíd, Aleš Hamáček, Stanislav Nešpůrek, Oldřich Zmeškal

**Affiliations:** 1 Department of Technologies and Measurement, Faculty of Electrical Engineering/RICE, University of West Bohemia, Plzeň 306 14, Czech Republic; E-Mails: xcaltsmid@fch.vutbr.cz (J.A.); hamacek@ket.zcu.cz (A.H.); nespurek@post.cz (S.N.); 2 Faculty of Chemistry, Brno University of Technology, Purkyňova 464/118, 612 00 Brno, Czech Republic; E-Mail: zmeskal@fch.vutbr.cz

**Keywords:** ionic liquid, amperometric NO_2_ sensor, carbon nanotube, aerosol-jet printing

## Abstract

A systematic study was carried out to investigate the effect of ionic liquid in solid polymer electrolyte (SPE) and its layer morphology on the characteristics of an electrochemical amperometric nitrogen dioxide sensor. Five different ionic liquids were immobilized into a solid polymer electrolyte and key sensor parameters (sensitivity, response/recovery times, hysteresis and limit of detection) were characterized. The study revealed that the sensor based on 1-ethyl-3-methylimidazolium bis(trifluoromethylsulfonyl)imide ([EMIM][N(Tf)_2_]) showed the best sensitivity, fast response/recovery times, and low sensor response hysteresis. The working electrode, deposited from water-based carbon nanotube ink, was prepared by aerosol-jet printing technology. It was observed that the thermal treatment and crystallinity of poly(vinylidene fluoride) (PVDF) in the solid polymer electrolyte influenced the sensitivity. Picture analysis of the morphology of the SPE layer based on [EMIM][N(Tf)_2_] ionic liquid treated under different conditions suggests that the sensor sensitivity strongly depends on the fractal dimension of PVDF spherical objects in SPE. Their deformation, e.g., due to crowding, leads to a decrease in sensor sensitivity.

## 1. Introduction

Room temperature ionic liquids (RTILs) are generally defined as the salts which are liquid under laboratory conditions [1]. They typically consist of a large and bulky organic cation (e.g., tetraalkyl-ammonium, pyrrolidinium, pyridinium, 1-alkyl-3-methylimidazolium) and an organic/inorganic anion (e.g., bis(trifluoromethylsulfonyl)imide, tetrafluoroborate, trifluoromethanesulfonate, or tris(pentafluoro-ethyl)trifluorophosphate). The big difference in the size of cations and anions does not allow the formation of an ionic lattice; instead, ions are usually disorganized, which results in the salts remaining liquid at room temperature [2]. RTILs have several archetypal properties, including very low volatility, a wide electrochemical window, high thermal stability, and quite good intrinsic conductivity [1,3,4,5]. The abovementioned properties and high structure modifiability make ionic liquids interesting and promising materials for use in fuel cells, batteries and electrochemical capacitors [6,7], in medicine [8,9], and in electrochemistry for the electrodeposition of various metals/semiconductors [6,10] and for extraction and separation techniques [11]. An interesting application is the use of RTILs for the electrochemical detection of various substances in gaseous or liquid phase [12,13,14,15,16]. In the recent past, electrochemical sensors based on different ionic liquids have been used for the detection of nitrogen dioxide [17], ammonia [17,18,19], oxygen [20,21], ozone [17] and ethylene [22]. Unfortunately, the use of ionic liquid in electronic devices requires a specific design and the fabrication of a micro-chamber for the storage of the ionic liquid electrolyte in order to prevent its leakage from the sensor package. This approach was used, e.g., by Stetter *et al.* [17,19,23], for the construction of ionic liquid-based amperometric sensors. Another approach to the preparation of gas sensors with ionic liquid is the use of solid polymer electrolyte (SPE) in which the ionic liquid is immobilized in the polymer matrix [24,25,26,27]. The experimental results described in this paper were obtained on this type of sensor for NO_2_ detection. Among other advantages, this approach allowed the fabrication of a low cost, fully printed, and flexible gas sensor on a PET substrate [27]. 

We immobilized five different ionic liquids in a blend of poly(vinylidene fluoride) (PVDF) and 1‑methyl-2-pyrrolidone (NMP). Key sensor parameters were determined and the ionic liquid with the best sensor sensitivity was used for the detailed study of the influence of electrolyte morphology on sensor sensitivity. A new technique, Aerosol Jet Printing (AJP), was used for the preparation of a working electrode, which allowed us to print an extremely thin active layer of carbon nanotubes (CNTs). 

## 2. Sensor Fabrication and Measurement Setup

### 2.1. Sensor Fabrication

**Figure 1:**
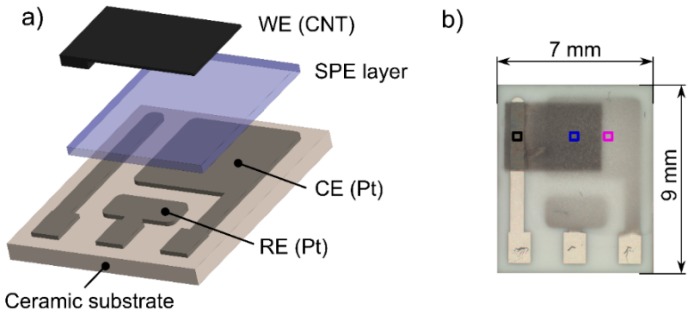
(**a**) Sensor layout; (**b**) Photograph of the nitrogen dioxide sensor (colored squares represent the areas captured in the SEM pictures (see Figure 5), black square (see Figure 2a), blue square (see Figure 2b), purple square (see in Figure 5).

The sensor platform was based on a well-established, three electrode topology that was successfully tested and thoroughly described in our previous works [26,27]. A ceramic substrate with platinum counter (CE) and pseudoreference (RE) electrodes (Figure 1a) was chosen as a reference electrode layout for the comparative study in which two key factors having an important impact on sensor characteristics were studied in detail: (i) the type of ionic liquid immobilized in the SPE; (ii) the structure and morphology of the electrochemical active layer at the interface between the SPE and the working electrode (WE). Therefore, the following subsections will be focused on the specification of the SPE formulations with different ionic liquids, a description of the procedures for the preparation of SPE layers with different morphologies, and a description of the new printing technology for the deposition of the working electrode.

#### 2.1.1. Solid Polymer Electrolyte

The solid polymer electrolytes (SPEs) in the present study were prepared in accordance with the procedure described in [26]. They consisted of a 1:1:3 weight ratio of an ionic liquid, poly-(vinylidene fluoride) (PVDF), and 1-methyl-2-pyrrolidone (NMP), where PVDF was used as polymer matrix and NMP as the solvent of the PVDF. Five different ionic liquids which had suitable properties (viscosity and thermal stability) for the formation of the SPE layer were immobilized in the PVDF matrix:

1. 1-ethyl-3-methylimidazolium bis(trifluoromethylsulfonyl)imide [EMIM][N(Tf)_2_];
2. 1-butyl-3-methylimidazolium trifluoromethanesulfonate [BMIM][CF_3_SO_3_];
3. 1-ethyl-3-methylimidazolium tetrafluoroborate [EMIM][BF_4_];
4. 1-hexyl-3-methylimidazolium tris(pentafluoroethyl)trifluorophosphate [HMIM][FAP];
5. 1-butyl-1-methylpyrrolidinium bis(trifluoromethylsulfonyl)imide [BMPYR][N(Tf)_2_].

All ionic liquids were used as received; [EMIM][BF_4_] and [HMIM][FAP] were obtained from Merck (Darmstadt, Germany), the others from Sigma-Aldrich (St. Louis, MO, USA). All sensors were prepared according to the following procedure: each SPE formulation with a particular ionic liquid was kept at 70 °C for 3 min to decrease its viscosity. Subsequently, 15 mg of the SPE formulation was drop-casted onto the ceramic substrate (Figure 1), which was then placed on a hot plate (120 °C for 3.5 min). Finally, before the last step of the fabrication process, *i.e.*, the deposition of the CNT working electrode by AJP technology, all samples were kept under laboratory condition for 24 h.

The morphology and structure of the SPE layers, which depend predominantly on the crystalline phases of PVDF, were studied by scanning electron microscopy and X-ray diffraction analyses. It is known that, depending on the crystallization conditions, PVDF forms at least four different well-known crystalline structures. Gregorio and Borges [28] described the influence of temperature on the crystallization rate and its effect on the formation of the different crystalline phases of PVDF dissolved in NMP. They also found a relationship between the porosity of the prepared layers and the crystallization temperature. It was observed that a higher crystallization temperature resulted in lower porosity. Thus, we also studied the influence of temperature and crystallization time on SPE morphology and porosity. For this experiment, only [EMIM][N(Tf)_2_] ionic liquid was taken into account due to the good film forming properties of SPE and the optimal parameters of the sensor (see Table 1, Section 4). In this case, the preparation procedure for the sensor was slightly modified. After the deposition of SPE onto a ceramic substrate, the sensor structure was kept at an appropriate temperature for a specific time. Four types of samples were prepared under different crystallization conditions: sample No. 1, thermal treatment condition 80 °C for 1.5 min; sample No. 2, 120 °C for 1.5 min; sample No. 3, 120 °C for 3.5 min and sample No. 4, 160 °C for 10 min. Then, the samples were kept under laboratory conditions for 24 h and photographs of SPE surfaces were taken by an electron microscope (Phenom-World, Eindhoven, The Netherlands). Picture analysis was carried out using the HarFa computer program [29]. Finally, the CNT working electrode was printed by the AJP technique.

#### 2.1.2. Deposition of the Working Electrode 

**Figure 2:**
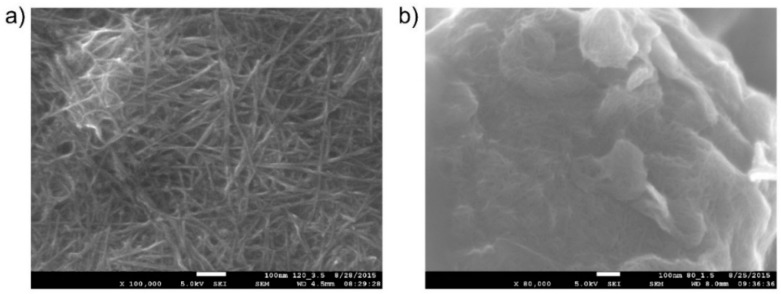
(**a**) Reference layer of carbon nanotubes on a flat surface (JEOL JSM 7600F microscope, magnification 100,000×); (**b**) Active SPE/WE interface layer (detail of one spherical object in the SPE layer covered by a thin layer of carbon nanotubes; thermal treatment conditions of the SPE layer, 80 °C for 1.5 min; JEOL JSM 7600F microscope, magnification 80,000×).

A new additive technology, Aerosol Jet Printing, was used for the deposition of the carbon nanotube working electrode (WE). This non-contact, direct-write printing process enables a wide variety of materials to be deposited onto a wide variety of substrates. With the use of computer-aided design/computer-aided manufacturing (CAD/CAM) software, AJP allows rapid prototyping in a large area of printed electronics [30]. For our purposes, this approach was utilized for effective optimization of the geometry, topology and morphology of the electrode. Moreover, this fabrication process significantly reduces ink consumption, which is important when suspensions of noble metals (e.g., Pt and Au) are considered for use as materials for the working electrode. We used surfactant-free, water-based carbon nanotube (CNT) ink (CNTRENE C100LM, Brewer Science, Rolla, MO, USA) for the printing of the working electrode. CNT sensing layers are generally used due to their unique material properties, high surface-to-volume ratio and hollow structure which is ideal for adsorption of gas molecules. We printed four CNT layers one over the other (area 10 mm^2^; at a total thickness of about 10–20 nm, the layer was transparent). The morphology of the electrode, obtained by a JSM 7600F electron microscope (JEOL, Peabody, MA, USA), is shown in Figure 2. Figure 2a shows the morphology of the layer of carbon nanotubes on a flat surface; Figure 2b shows the active SPE/WE interface (the detail of one spherical object in the SPE layer covered by a thin layer of carbon nanotubes). Thus, the use of AJP technology enabled a detailed picture of the electrochemical active interface to be made (due to the thinness and transparency of the working electrode). It should be noted that the quality, structure, and morphology of the working electrode and its interface with SPE strongly influence sensor parameters.

### 2.2. Measurement Setup

The gas test system for the measurement of sensor characteristics consisted of two gas tanks (the first filled with a reference mixture of gaseous nitrogen dioxide [100 ppm NO_2_] balanced with synthetic air; the second filled with pure synthetic air), five PC-controlled mass flow controllers, and a test chamber which enabled the measurement of six sensors within one experiment. The characteristics of all sensors were measured under identical conditions (unless otherwise stated: 22 °C, 40% RH, analyte flow rate = 500 mL/min). A six-channel evaluation board with configurable analog front end (AFE) potentiostats (LMP91000, Texas Instruments, Dallas, TX, USA) was used as the electronics read-out. The output analog voltage level from each potentiostat was sampled every other second by two four-channel 24‑bit analog to digital converters ADC (LTC2493, Linear Technology, New York, NY, USA) and data were subsequently transferred to the PC via a USB bus. The optimal bias voltage, −500 mV*∙vs.* the platinum pseudoreference electrode, was obtained from a steady state polarization curve [27]. Even though the potential stability of the platinum pseudo/quasi-reference electrode is unknown, it has been demonstrated that such sensor can exhibit good response stability during a long term test [26]. 

## 3. Results and Discussion

### 3.1. Effect of the Type of Ionic Liquid

**Figure 3:**
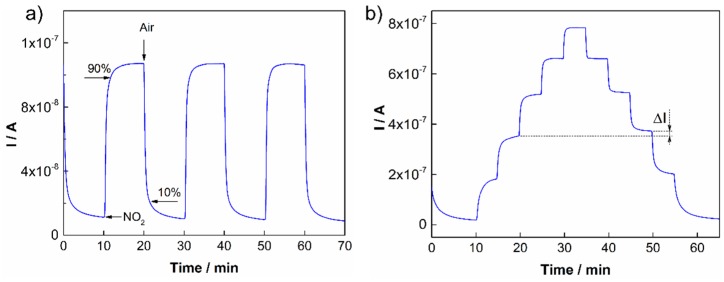
(**a**) Sensor response to repeated exposures to 1 ppm NO_2_ [BMPYR][N(Tf)_2_]; (**b**) Sensor response to stepwise increases in NO_2_ concentrations: 1, 2, 3, 4 and 5 ppm NO_2_ [EMIM][N(Tf)_2_].

The detection of nitrogen dioxide by amperometric sensors with polymer electrolytes is relatively well known and particular redox reactions on the working and counter electrodes have been described in the literature [31,32]. This work is focused on the systematic study concerning key sensor parameters for the five ionic liquids mentioned above. The parameters are summarized in Table 1. All sensors with different ionic liquids intercalated into SPE were prepared in accordance with the procedure described in Section 2.1.1. Each SPE layer with a particular ionic liquid was formed under the same thermal conditions (120 °C for 3.5 min) in order to eliminate possible effects of the thermal conditions on sensor characteristics. Subsequently, sensors were tested under repeated (Figure 3a) and stepwise increases/decreases in NO_2_ exposure (Figure 3b). In the first case, the NO_2_ concentration was 1 ppm. From the representative Figure 3a (for the ionic liquid [BMPYR][N(Tf)_2_]), it follows that the stability and reproducibility of the sensor response was very good. 

**Table 1:** Sensor parameters.

Ionic Liquid	Sensitivity (pA/ppb)	Response/Recovery Time * (s)	Hysteresis ΔI (%)	LOD (ppb)
[EMIM][N(Tf)_2_]	149	(53 ± 3)/(68 ± 3)	7	0.9
[BMIM][CF_3_SO_3_]	48	(70 ± 4)/(74 ± 4)	5	2
[EMIM][BF_4_]	59	(64 ± 3)/(90 ± 3)	5	1.6
[HMIM][FAP] **	---	(43 ± 3)/(38 ± 3)	42	---
[BMPYR][N(Tf)_2_]	91	(46 ± 3)/(44 ± 3)	13	0.3

* Average values with 99% confidence interval was calculated from five consecutive exposures; ** Sensitivity and LOD of the sensor were not determined because of the non-linear calibration curve resulting from the instability of the sensor response (Figure 3a).

**Figure 4:**
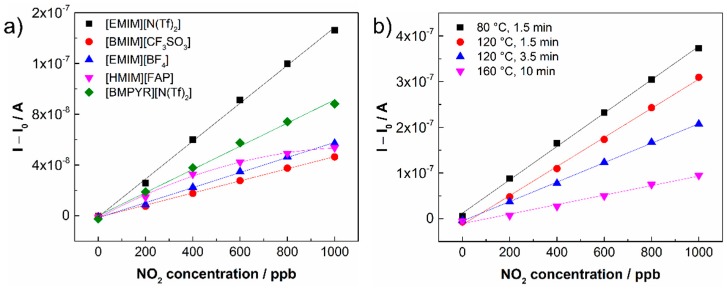
(**a**) Dependence of sensor current on NO_2_ concentration for different ionic liquids in SPE; (**b**) Dependence of sensor current on NO_2_ concentration for different thermal treatment conditions during the formation of the SPE layer with [EMIM][N(Tf)_2_] ionic liquid: sample No. 1, 80 °C for 1.5 min; sample No. 2, 120 °C for 1.5 min; sample No. 3, 120 °C for 3.5 min and sample No. 4, 160 °C for 10 min.

The response/recovery time and hysteresis of the sensor response were determined according to the usual rules. The response time is defined as the time necessary for the increase in sensor response to reach 90% of its steady state value under analyte exposure. The recovery time is determined as the time necessary for the decrease in sensor response to reach the 10% level when the sensor is exposed to pure air (see Figure 3a). The hysteresis of the sensor response was determined as the difference between the output currents for the same NO_2_ concentration during increasing and decreasing exposures (Figure 3b). The maximum difference was related to the upper limit of the measured current and expressed as a percentage (Table 1). The other parameters, the sensitivity and the theoretical limit of detection (LOD), were calculated from the calibration curves of the particular sensors (Figure 4a). The sensitivity was determined as the slope of the calibration curve, and the LOD was taken to be equal to 3 rms_noise_/slope, where rms_noise_ is the root-mean-square noise of the baseline. It should be noted that the actual LOD values may differ from those calculated according to the abovementioned expression because the activation of the working electrode surface (or the interface SPE/WE) may require a minimal amount of analyte. The sensitivity of sensors was studied within a low concentration range from 0 to 1 ppm (Figure 4a), where the NO_2_ concentration was increased in the following steps: 200, 400, 600, 800 and 1000 ppb. The calibration curves of the sensors in Figure 4 were compared using a normalization approach, where *I* represents the current response of the sensor under analyte exposure and *I*_0_ is the “background current” when the sensor is exposed to pure air. Table 1 provides an overview of the key sensor parameters for all tested ionic liquids. 

The highest sensitivity was found for the sensor based on [EMIM][N(Tf)_2_] ionic liquid, the lowest LOD was obtained for the sensor based on [BMPYR][N(Tf)_2_] (due to the lowest rms_noise_ of the background current). Response/recovery times were in the order of tens of seconds for all sensors. The levels of hysteresis were acceptable for all tested sensors except the sensor based on [HMIM][FAP] ionic liquid, whose value was influenced by the instability of the sensor response. The sensor based on [EMIM][N(Tf)_2_] ionic liquid was selected for further detailed study because it exhibited the highest sensitivity, a fast response/recovery time, and an acceptable hysteresis level, and because of the good film-forming properties of the SPE formulation in terms of the absence of defects in the SPE layer after thermal treatment and good adhesion to the ceramic substrate.

### 3.2. Surface Activity of the Sensor

**Figure 5:**
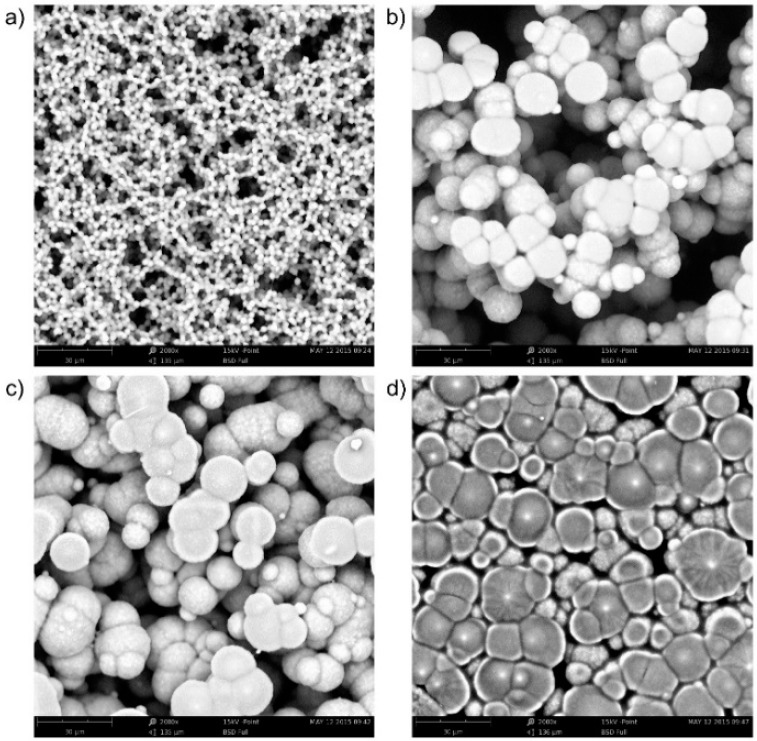
Morphology of the SPE layer based on [EMIM][N(Tf)_2_] ionic liquid under different thermal treatment conditions (Phenom ProX electron microscope, magnification 2000×): (**a**) 80 °C for 1.5 min; (**b**) 120 °C for 1.5 min; (**c**) 120 °C for 3.5 min; (**d**) 160 °C for 10 min.

To obtain some information on the relation between sensitivity and film morphology, picture analysis of the morphology of thermally treated SPE layers was performed. Four pictures were taken into account (Figure 5; the treatment conditions are given in Section 2.1.1. From Figure 4b, it follows that the sensitivity of the sensor depends on the temperature treatment of the SPE layer. The morphology of the layers also changes during thermal treatment (see Figure 5). The surface of the most sensitive layer (Figure 5a, treatment 80 °C, 1.5 min) consists of very small spherical SPE objects, the active surface is large, and the surface structure is very ragged. In contrast, the surface of the least sensitive layer (Figure 5d, treatment 160 °C, 10 min) consists of relatively large deformed spherical objects. Both the active surface is much smaller and the complexity of the edge structure is significantly reduced.

In what follows, we try to find the link between the size and deformation of the objects and sensor sensitivity using the method of fractal image analysis. Using the fractal parameters fractal measure and fractal dimension, it is possible to determine the active surface area and its complexity of edge structure (topological entropy), respectively [33,34]. The HarFA 5.1 computer program [29] was used to calculate the fractal parameters. The program makes it possible to determine surface areas in planes of different depths from the SPE surface (areas of contour lines) and the complexity of their edge structure at these depths using the method of box counting, which is based on wavelet analysis (the Haar transformation) [35]. The Haar transformation is an integral transformation with a base of “square-shaped” functions. Wavelet analysis is similar to Fourier analysis in the sense that it allows a target function over an interval to be represented in terms of an orthonormal function basis. The principle of the wavelet method is based on the screening of black and white images. The screening can be generally performed by low level filtering; in the case of the Haar transformation, it can be calculated simply by averaging the nearest four pixels (or squares of screened image). 

The parameters mention above, *i.e.*, surface areas in planes of different depths and their complexity influence the activity of the SPE surface. The calculation of the parameters by the box counting method is based on the known expression:

(1) $$N=K\;r^{D}$$

where *N* is the number of pixels, *K* is the fractal measure, *r* is the size of squares (in pixels) and *D* is the fractal dimension. This equation expresses the dependence of the number of squares with defined properties (*i.e.*, whether they are all black, all white, or contain both black and white pixels) in the threshold image on their dimension. From the logarithmic dependence of Equation (1) for various sizes of squares (1 × 1, 2 × 2, 4 × 4, 8 × 8, …, pixels), we are able to use a linear regression to determine both fractal parameters—fractal measure *K* and fractal dimension *D*—for all cases with three independently defined properties (BBW—black area including interface, WBW—white area including interface, and BW—area of interface). From the fractal measures *K*_BBW_, *K*_WBW_, and *K*_BW,_ the relative areas *A*_B_ (number of pixels in the black area), *A*_W_ (number of pixels in the white area), and *A*_BW_ (number of pixels in contour line) can be calculated according to the expressions:

(2) $$A_{B}=\frac{K_{\text{BBW}}-K_{\text{BW}}}{K_{\text{BBW}}+K_{\text{WBW}}-K_{\text{BW}}}\;,A_{W}=\frac{K_{\text{WBW}}-K_{\text{BW}}}{K_{\text{BBW}}+K_{\text{WBW}}-K_{\text{BW}}},\;\;A_{\text{BW}}=\frac{K_{\text{BW}}}{K_{\text{BBW}}+K_{\text{WBW}}-K_{\text{BW}}}$$

These areas are usually normalized to 1, *i.e.*, *A*_B_ + *A*_W_ + *A*_BW_ = 1. The maximal fractal measure has the value of *K*_max_ = K_B_ + K_W_ + K_BW_ = (1024 × 1024) pixels.

A similar procedure can be used to determine the relation between Hausdorff fractal dimensions and the complexity of the surface structure expressed by means of topological entropies:

(3) $$S_{\text{BBW}}=D_{\text{BBW}}\ln r,S_{\text{WBW}}=D_{\text{WBW}}\ln r,\;S_{\text{BW}}=D_{\text{BW}}\ln r$$

or by their deviations from a plane surface (D = 2). Here, D_BBW_, D_WBW_, and D_BW_ are the fractal dimensions of the black area including interface, the white area including interface, and the area of interface, respectively.

**Figure 6:**
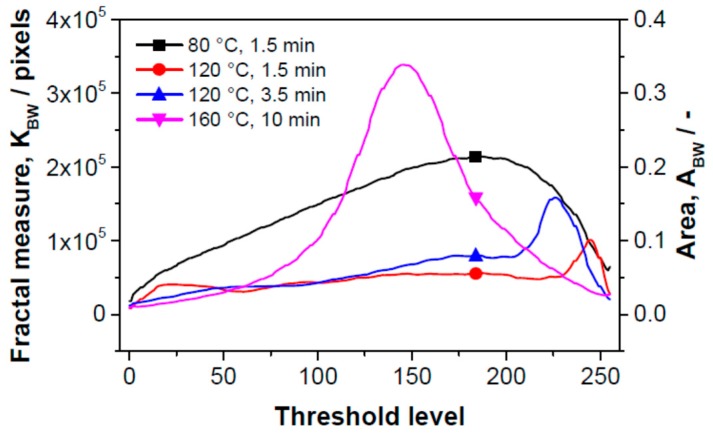
Dependence of the fractal measure of the surface between contour lines (threshold levels) on the magnitude of the threshold of gray scale images for sensors with differently thermally treated SPE layers.

**Figure 7:**
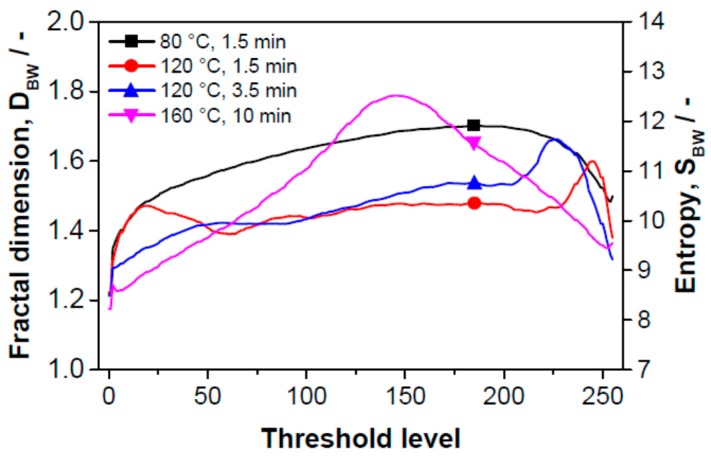
Dependence of the fractal dimension of the boundary of threshold images (resp. surface inhomogeneity expressed as topological entropy) on the threshold level of gray scale images.

Two-dimensional image analysis as a way of measuring surface area was applied to a 3‑dimensional material as follows: The black place in microscopic image was taken as zero level (unfortunately we have no information about the actual deepness of this level). The space bounded by “black” plane and sensor surface (the whitest places in the microscopic image) was cut into 250 slices. Their positions are given in Figure 6 and Figure 7 as threshold levels, the deepest level is positioned to zero (black place in microscopic image). The total area (ABW) of parts of spherical objects visible from the direction of the sensor surface, sum of the circumferences of spherical objects (relief divison) are determined in each slide. Thus, fractal dimension on the position of the threshold level (*i.e.*, in 3-dimensional space) is known as well. Counting up the areas we can determine the total “active surface area” of the sensor and its entropy.

Figure 6 gives the dependence of the fractal measure of the surface between contour lines (between threshold levels) on the threshold level of the “gray scale” image. The threshold level is the position of the contour line; it is defined as “0” in the bulk of the SPE layer—the maximal depth accessible by microscope (black color); “255” means the surface of SPE layer. The dependence of fractal measure on threshold level is influenced by two parameters: (i) by the active area of the sectional plane which influences the sensitivity of the sensor at the respective distance from the electrolyte surface (the distance in absolute units is not presented here); (ii) by the area which characterizes the deformation of the objects–this contribution decreases the sensor sensitivity. It should be noted that the fractal measure is positive in both cases. Each curve in Figure 6 contains both contributions with different benefits. No deformation of objects was observed for the sample in which SPE was treated at 80 °C for 1.5 min (structure (a) in Figure 5, black curve in Figure 6).

Here, the fractal measure is high and therefore the sensor is highly sensitive. Concerning the sensitivity profile of SPE, the most sensitive “layer” is located at the distance (threshold level) “180” (the 180th contour line from the maximal depth accessible by microscope; the black color on the microscopic image). At this depth the active sensor area is the greatest.

By contrast, the deformation contribution is dominant for the sample in which SPE was treated at 160 °C for 10 min (structure (d) in Figure 5, purple curve in Figure 6) and the sensitivity of the sensor is low. The deformation depth is extended up to about 40% of the “visible” depth. The maximum of the deformation activity is at the threshold level “145”, *i.e.*, deeper than the maximum of the sensitivity for the sample which was thermally treated at 80 °C for 1.5 min. Both active (sensitivity) and deformation contributions are visible for the sensors in which the thermal treatment of SPE was carried out at 120 °C for 1.5 min (structure (b) in Figure 5, red curve in Figure 6) and at 120 °C for 3.5 min (structure (c) in Figure 5, blue curve in Figure 6) . The threshold levels with higher deformation activities are located near the sensor surface; the maxima of the “deformation” peaks are positioned at the threshold level “245” (red curve) and “225” (blue curve). The threshold active levels with the highest “sensitivity” have a position at about “180”. Thus, the highest sensitivity of the sensor is not at the SPE/working electrode boundary but at a certain distance from the SPE surface. From this distance to the SPE surface, the “deformation areas” occur, *i.e.*, regions where spherical objects of SPE are deformed or form aggregates or crowds. Taking into account both sensitivity and deformation contributions, the calculated progression of the sensitivity agrees well with experimental results. Another effect which influences the overall sensor sensitivity is the penetration depth of NO_2_ molecules; however, this effect is not discussed in this paper. 

Figure 7 shows the dependence of the fractal dimension of the boundary of the threshold images corresponding to the edge relief of the surface expressed as topological entropy on the threshold level. The conclusions from this figure are the same as those following from Figure 6.

**Figure 8:**
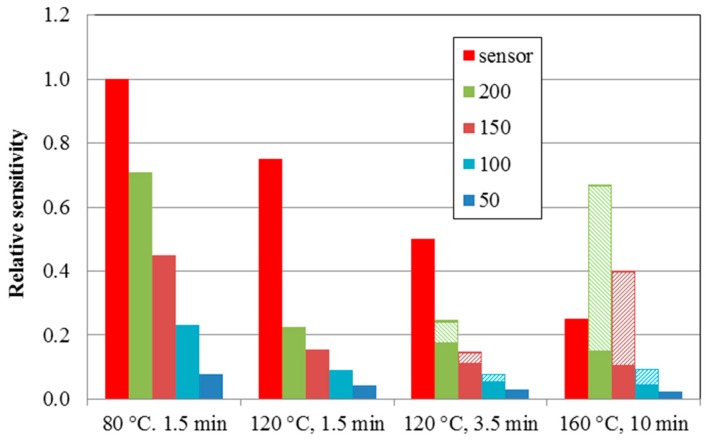
Experimental relative sensitivities for structures from Figure 5 (red columns). Calculated relative active (sensitivity) areas (full columns) and deformation contribution areas (hatched columns)—here marked as “relative sensitivity”. Differently colored columns indicate the contributions below the defined threshold level (“50”—dark blue columns, “100”—light blue columns, “150”—brown columns and “200”—green columns). Note that “255” represents the surface of the SPE layer.

Figure 8 summarizes the experimental and theoretical results presented in this work. It shows the experimental relative sensitivities for the structures from Figure 5 (red columns). The sensor sensitivity decreases with treatment at higher temperatures (see Figure 4b). The calculated relative active areas (full columns) below the defined threshold levels (“50”—dark blue columns, “100”—light blue columns, “150”—brown columns, and “200”—green columns) agree well with the experimental progression of the sensor sensitivity. The thermal treatment of the sensor leads to the deformation, clustering, and crowding of ball-shaped SPE objects, increases the deformation contribution (hatched columns), and decreases the sensor sensitivity. This work revealed that the deformation effect starts at a temperature treatment of 120 °C for 3.5 min and increases with both higher temperature and longer heating time.

## 4. Conclusions

A systematic study of an electrochemical NO_2_ sensor with an ionic liquid-based solid polymer electrolyte and an aerosol jet printed carbon nanotube working electrode was performed. The sensor with the ionic liquid 1-ethyl-3-methylimidazolium bis(trifluoromethylsulfonyl)imide ([EMIM][N(Tf)_2_]), exhibited the highest sensitivity. The basic parameters were as follows: sensitivity—150 pA/ppb; response/recovery time—53/68 s; hysteresis ΔI—7%; and theoretical limit of detection—0.9 ppb. The lowest sensitivity (from the set of five ionic liquids) was exhibited by the sensor with the ionic liquid 1-butyl-3-methylimidazolium trifluoromethanesulfonate ([BMIM][CF_3_SO_3_]); this was found to be 48 pA/ppb. Thermal treatment was found to decrease sensor sensitivity; the higher the temperature and the longer the time of heating, which increases the deformation and crowding of PVDF ball-shaped objects, the lower the sensitivity. Picture analysis of the morphology of the SPE layers suggests that sensor sensitivity strongly depends on the fractal dimension of PVDF objects in SPE. The most sensitive sensor, based on [EMIM][N(Tf)_2_] ionic liquid (thermal treatment at 80 °C, 1.5 min), exhibited the greatest area of PVDF ball objects (sensitive area) and no deformation area. The fractal measure amounted to 2 × 10^5^ pixels. Thermal treatment at 160 °C for 10 min resulted in a sensor with a sensitivity 3.5 times lower and a large integrated area of deformed objects. Independently of thermal treatment, the highest contribution to sensitivity was not caused by the surface layer of the SPE, *i.e.*, at the SPE/working electrode interface, but by deeper “layers” in the bulk.
